# 

**Published:** 1892-08

**Authors:** 


### LITERARY.

THE WIFE AND MOTHER : A MEDICAL GUIDE TO THE CARE OF HER HEALTH AND THE MANAGEMENT OF HER CHILDREN.—By Albert Westland, M.A., M.D., C. M. P. Blakiston, Son & Co., 1012 Walnut street, Philadelphia, Publishers. A 4to of 275 pp., price $2.00.

A timely volume, replete of information which every maid, wife and mother should possess. Nearly all the ailments that flesh is heir to, from the cradle to the grave, are diagnosed, explained and treated of in so plain and comprehensive a manner that a mistake is quite out of reason.

As a FAMILY MEDICAL GUIDE, complete in all its parts, it will be found without a peer. The chapters on the care and treatment of children alone are worth more than the price of the book and yet they form only a considerable share of it.

No head of a family can invest two dollars more profitably than by sending for it.

OUR LITTLE MEN AND WOMEN FOR AUGUST. Price $1.00 a year, 10 cents a number. D. Lothrop Co., Publishers, Boston.

Gives the boys a hint which they will understand in "What the Brook Saw." Joker, the clever monkey, meets some remarkably clever relatives, and "What Spoiled the Day," "Dick's Auction," "A Little Builder," "The Plague of Locusts," "A Boy and a Girl," "Talks by Queer Folks," "Did Tabby Understand?" "The Tally-ho Jaunt," are among the bright stories by bright writers, who with the clever artists help to make this publication the best and brightest boys' and girls' magazine ever issued.

We are advised that Mr. Henry Sell, of London, Editor of Sell's Dictionary of the World's Press, has been awarded space for a Newspaper Exhibition at the World's Fair. This is a most worthy undertaking and we trust it may not come short of the expectations of its projector.

